# No evidence for a direct extracellular interaction between human Fc receptor–like 3 (MAIA) and the sperm ligand IZUMO1

**DOI:** 10.1126/sciadv.adk6352

**Published:** 2024-02-21

**Authors:** Enrica Bianchi, Maria Jiménez-Movilla, Paula Cots-Rodríguez, Cristina Viola, Gavin J. Wright

**Affiliations:** ^1^ Department of Biology, Hull York Medical School, York Biomedical Research Institute, University of York, York, UK.; ^2^ Department of Cell Biology and Histology, Medical School, University of Murcia, Instituto Murciano de Investigación Biosanitaria (IMIB-Arrixaca), Murcia, Spain.

## Abstract

Fertilization involves the recognition and fusion of sperm and egg to form a previously unidentified organism. In mammals, surface molecules on the sperm and egg have central roles, and while adhesion is mediated by the IZUMO1-JUNO sperm-egg ligand-receptor pair, the molecule/s responsible for membrane fusion remain mysterious. Recently, MAIA/FCRL3 was identified as a mammalian egg receptor, which bound IZUMO1 and JUNO and might therefore have a bridging role in gamete recognition and fusion. Here, we use sensitive assays designed to detect extracellular protein binding to investigate the interactions between MAIA and both IZUMO1 and JUNO. Despite using reagents with demonstrable biochemical activity, we did not identify any direct binding between MAIA/FCRL3 and either IZUMO1 or JUNO. We also observed no fusogenic activity of MAIA/FCRL3 in a cell-based membrane fusion assay. Our findings encourage caution in further investigations on the role played by MAIA/FCRL3 in fertilization.

## Teaser

A reassessment of the role of MAIA/FCRL3 in fertilization reveals no direct extracellular binding to IZUMO1.

## INTRODUCTION

Fertilization occurs when male sperm recognizes and fuses with female eggs to create a new genetically distinct individual (*1*). While the core genetic principles of sexual reproduction are conserved across the different kingdoms of life, the molecules used by different species are remarkably diverse impeding their discovery. This is particularly true because both sperm and eggs are terminally differentiated cells that do not divide, and there are understandable ethical restrictions on studying human fertilization in vitro. These and other technical challenges have made identifying the molecules involved in mammalian fertilization difficult (*2*).

The development of genetic targeting in mice has been instrumental in much of the recent progress in discovering the molecules important for fertilization in mammals and led to a critical reassessment of many proteins that were initially thought to be involved (*3*). The central role of the sperm ligand IZUMO1 emerged from these studies because it was shown that male mice with a targeted disruption of the *Izumo1* gene were infertile despite producing morphologically normal sperm (*4*). IZUMO1 is a type I membrane protein that is sequestered internally within the acrosome of intact sperm that redistributes to the exposed equatorial region of the sperm head once the acrosome reaction is complete (*5*). An egg binding partner for IZUMO1 named JUNO was identified, and the importance of this interaction was demonstrated by showing that female *Juno*-deficient mice, which produced apparently normal eggs, were also infertile (*6*). Structural studies of the IZUMO1:JUNO complex have shown that IZUMO1 contains a four-helix bundle at its N terminus and a membrane-proximal immunoglobulin (Ig)–like domain, and the globular JUNO protein binds at the junction of these two domains (*7*, *8*). The rapid shedding of the JUNO protein from the egg membrane after fertilization also provided a plausible molecular explanation for the membrane block to polyspermy (*6*, *9*). The essential role of IZUMO1:JUNO in human fertilization has also been confirmed (*1*, *10*, *11*).

Despite this progress, there is much that remains to be understood about the molecular events at fertilization and especially the identity of a “fusogen”: a protein or proteins that are required to fuse the sperm and egg membranes (*12*). It does not appear that the IZUMO1-JUNO interaction has a direct role in membrane fusion because neighboring cells expressing these proteins do not fuse (*6*), nor do IZUMO1-expressing cells fuse with oocytes (*13*–*15*), suggesting that this interaction has an adhesive rather than fusogenic role. Targeted gene deletions in mice have more recently identified a range of other cell surface and secreted proteins that are essential for mammalian fertilization, including the sperm proteins TMEM95 (*15*, *16*), SPACA6 (*17*, *18*), SOF1 (*15*), FIMP (*19*), DCST1, DCST2 (*20*, *21*), TMEM81 (*22*), and egg CD9 (*23*, *24*) [reviewed in (*25*)]. Biochemical and modeling studies have suggested that some of these proteins may function as part of a larger multiprotein complex (*22*, *26*); however, whether this complex is directly involved in membrane fusion is not yet clear. Recent research has suggested that IZUMO1 could itself directly act as a membrane fusogen (*27*), although some questions remain (*28*). Other clues to the existence of a fusogenic protein were obtained from cell biological studies which suggested that structural rearrangements of IZUMO1, which were dependent on JUNO binding, reveal an interaction surface for an additional but unidentified IZUMO1 receptor on eggs (*29*).

More recently, a previously unidentified interaction between Fc receptor–like protein 3 (FCRL3, renamed MAIA by the authors) and IZUMO1 was reported (*30*). FCRL3/MAIA was initially identified because of sequence matches with a six–amino acid peptide immobilized on a bead that bound sperm and was prevented by preincubating sperm with an anti-IZUMO1 antibody. Like IZUMO1, FCRL3 is a cell surface protein anchored to the membrane by a single transmembrane–spanning region and also belongs to the Ig superfamily of proteins, containing six extracellular Ig-like domains, but unlike *IZUMO1* and *JUNO*, the *FCRL3* gene is not conserved in mice, where only a paralog is present (*31*). The study additionally showed associations between FCRL3/MAIA and JUNO, suggesting that it could act as an important bridge between the sperm and egg membranes. Because of its potentially important role in fertilization and membrane fusion, here, we have investigated the extracellular interactions of FCRL3 with IZUMO1. Using a range of experimental approaches, which are designed to detect low-affinity extracellular protein interactions (*32*, *33*), we report that we are unable to detect any direct interactions between FCRL3 and either IZUMO1 or JUNO.

## RESULTS

**Fig. 1.**
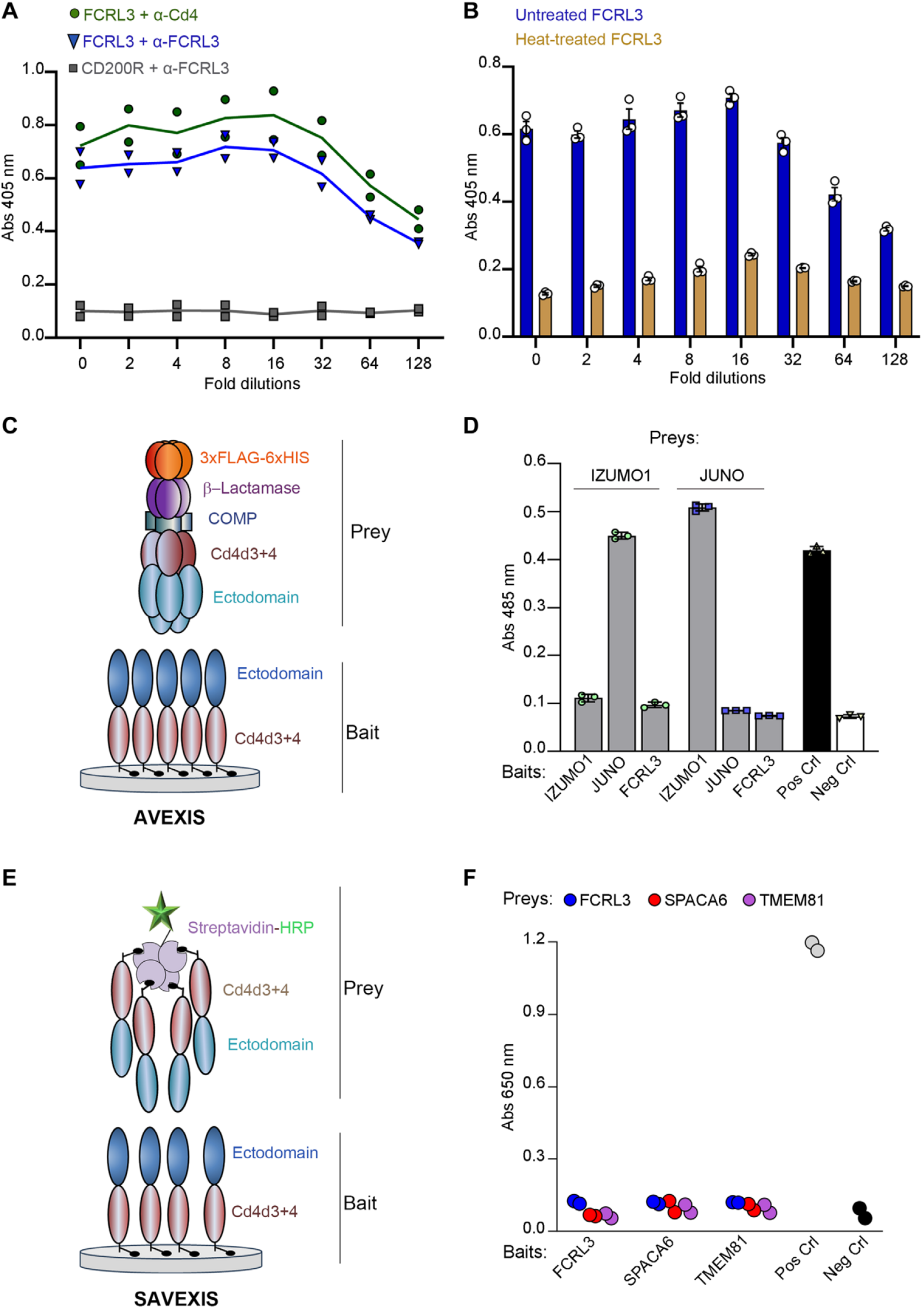
No evidence for a direct interaction between FCRL3 and IZUMO1. (**A**) The entire biotinylated ectodomain of FCRL3 was expressed as a rat Cd4–tagged fusion protein, serially diluted and captured in wells of a streptavidin-coated microtiter plate. Protein capture was confirmed using an anti-FCRL3 antibody MAB3126 (blue triangles) and anti-rat Cd4 antibody (green circles) by ELISA. Rat CD200R ectodomain was used as a negative control (gray squares). Data points represent duplicate values. (**B**) FCRL3 ectodomain contains conformational epitopes. Serial dilutions of biotinylated FCRL3 ectodomain were captured on streptavidin-coated plates after heat treatment (80°C for 10 min, orange bars) or untreated (blue) and immunoreactivity to anti-FCRL3 antibody MAB3126 quantified by ELISA. The loss of immunoreactivity upon heat treatment demonstrated the presence of conformational epitopes. Data points show triplicate readings ± SD. (**C**) Schematic of the AVEXIS assay. (**D**) FCRL3 does not interact with IZUMO1 or JUNO. Highly avid β-lactamase–tagged IZUMO1 and JUNO proteins were tested for direct binding to the named bait proteins using the AVEXIS assay. No binding was observed to FCRL3 with either the IZUMO1 or JUNO prey proteins. Controls were the rat CD200 prey binding CD200R bait (positive) or rat Cd4 (negative). Data points are in triplicate ± SD. (**E**) In the SAVEXIS assay, baits and preys are expressed as monomeric biotinylated ectodomains, purified, and quantified. Multimeric preys are produced by clustering biotinylated monomers around streptavidin-HRP and probed against baits arrayed on streptavidin-coated plates. (**F**) FCRL3 does not interact with the essential sperm surface proteins SPACA6 or TMEM81 by SAVEXIS. Positive control was human JUNO (bait) and human IZUMO1 (prey); negative control was FCRL3 (bait) with no preys. These are representative data from two [(A), (B), and (F)] and three (D) independent experiments.

To further investigate the ability of FCRL3/MAIA to interact with IZUMO1, we first expressed the entire extracellular domain of FCRL3 as a soluble recombinant enzymatically biotinylated protein in mammalian cells (Table 1). We demonstrated that the FCRL3 ectodomain was expressed by enzyme-linked immunosorbent assay (ELISA) and bound by a monoclonal antibody to FCRL3 (clone MAB3126), the same antibody used by Vondrakova *et al.* (*30*) (Fig. 1A). We demonstrated that the FCRL3 ectodomain was folded correctly by showing the loss of antibody binding after heat treatment (Fig. 1B). To determine whether FCRL3 could directly interact with IZUMO1, we used an assay called AVEXIS (avidity-based extracellular interaction screening), which is specifically designed to detect extracellular receptor ligand interactions that are often characterized by weak binding affinities (Fig. 1C) (*32*, *34*). This assay quantifies direct binding between receptor ectodomains presented as biotinylated “baits” captured on a streptavidin-coated plate and highly avid enzyme-tagged “preys” and can detect interactions that have equilibrium dissociation constants (*K*_D_s) in the high micromolar range (*32*). Using this approach, we were able to detect direct interactions between human IZUMO1 and JUNO (Fig. 1D); however, no direct interactions between FCRL3/MAIA and either IZUMO1 or JUNO could be detected using this approach. IZUMO1 has recently been shown to form a complex with two structurally related sperm cell surface proteins: SPACA6 and TMEM81 (*22**,* *26*). Both mice and zebrafish with targeted deletions in any one of these three genes results in male sterility (*4*, *15*, *17*, *18*, *22*). To determine whether FCRL3/MAIA could bind to either SPACA6 or TMEM81, we used a modified version of the AVEXIS assay called SAVEXIS (Fig. 1E) (*35*); again, we were unable to detect any direct interactions with FCRL3/MAIA (Fig. 1F).

**Table 1.** List of expression constructs.

Gene name (species)	Protein ID ectodomain truncation residue	Tags (at the 3′ end of the gene sequence)
*IZUMO1* (human)	Q8IYV9 P285	Domains 3 and 4 of rat CD4, biotinylation target sequence, 6*HIS (bait)
		Domains 3 and 4 of rat CD4, COMP, β-lactamase, FLAG, 6*HIS (prey)
		Transmembrane domain of rat CD200R, EGFP (TM-GFP)
*JUNO* (human)	A6ND01 P231	Domains 3 and 4 of rat CD4, biotinylation target sequence, 6*HIS (bait)
		Domains 3 and 4 of rat CD4, COMP, β-lactamase, FLAG, 6*HIS (prey)
		Transmembrane domain of rat CD200R, EGFP (TM-GFP)
*FCRL3* (human)	Q96P31 T573	Domains 3 and 4 of rat CD4, biotinylation target sequence, 6*HIS (bait)
		Domains 3 and 4 of rat CD4, COMP, β-lactamase, FLAG, 6*HIS (prey)
		Transmembrane domain of rat CD200R, EGFP (TM-GFP)
		Transmembrane domain of rat CD200R, mCHERRY (TM-mCHERRY)
*SPACA6* (human)	W5XKT8 L295	Domains 3 and 4 of rat CD4, biotinylation target sequence, 6*HIS (bait)
*TMEM81* (human)	Q6P7N7 S226	Domains 3 and 4 of rat CD4, biotinylation target sequence, 6*HIS (bait)
*Cd4* (rat)	P05540 S209-N392	Domains 3 and 4 of rat CD4, biotinylation target sequence, 6*HIS (bait)
*Cd200R* (rat)	Q9ES58 G233	Domains 3 and 4 of rat CD4, biotinylation target sequence, 6*HIS (bait)
*Cd200* (rat)	P04218 K231	Domains 3 and 4 of rat CD4, COMP, β-lactamase, FLAG, 6*HIS (prey)
*Syncytin-A* (mouse)	Full-length cDNA (clone MC219753, OriGene)	Untagged

**Fig. 2.**
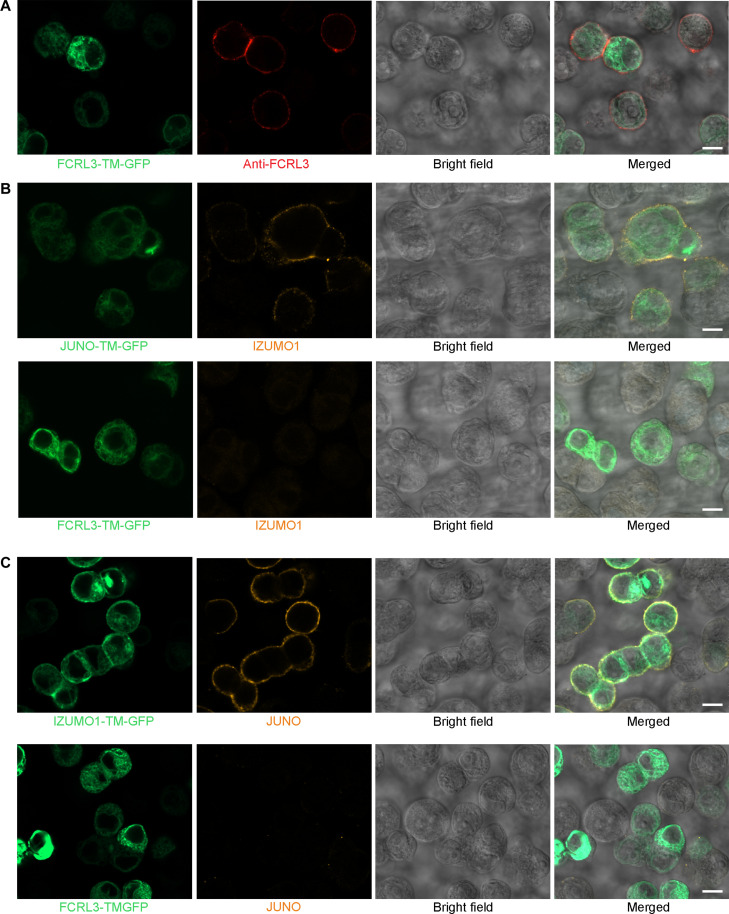
No evidence of FCRL3 binding to IZUMO1 and JUNO using a cell-based binding assay. (**A**) HEK293 cells were transfected with a plasmid encoding the entire ectodomain of FCRL3, a transmembrane spanning region and cytoplasmic GFP, fixed, but not permeabilized, and stained with an anti-FCRL3 monoclonal antibody (red). Only GFP-positive cells are stained with the anti-FCRL3 antibody demonstration cell surface presentation of the FCRL3 ectodomain and specificity of the antibody. (**B**) A highly avid fluorescent IZUMO1 binding probe made by clustering biotinylated monomeric IZUMO1 around a streptavidin-phycoerythrin conjugate and presented to HEK293 cells transfected with either JUNO-TM-GFP (top) or FCRL3-TM-GFP (bottom). (**C**) The JUNO ectodomain was presented as a pentameric binding probe and presented to HEK293 cells transfected with either IZUMO1-TM-GFP (top) or FCRL3-TM-GFP (bottom). In both (B) and (C), the IZUMO1-JUNO interaction could be detected, but no binding to FCRL3 was observed with either IZUMO1 or JUNO binding probes. Scale bars, 10 μm.

To further investigate whether FCRL3 could bind IZUMO1 or JUNO in the context of an intact cell membrane, we first fused the gene encoding the entire ectodomain of FCRL3 in frame to a mammalian expression plasmid containing a transmembrane region and cytoplasmic green fluorescent protein (GFP) (*36*). This cell-based assay was previously used to confirm the IZUMO1-JUNO interaction (*6*), which was successfully used as a control interaction here. We first verified that the ectodomain of FCRL3 was localized on the cell surface by demonstrating that unpermeabilized cells transfected with the plasmid encoding the FCRL3-TM-GFP protein were stained with the anti-FCRL3 antibody (Fig. 2A). Using either IZUMO1 or JUNO binding probes that demonstrated positive binding to cells transfected with their corresponding binding partner, we did not observe any positive staining to cells expressing cell surface FCRL3 (Fig. 2, B and C).

**Fig. 3.**
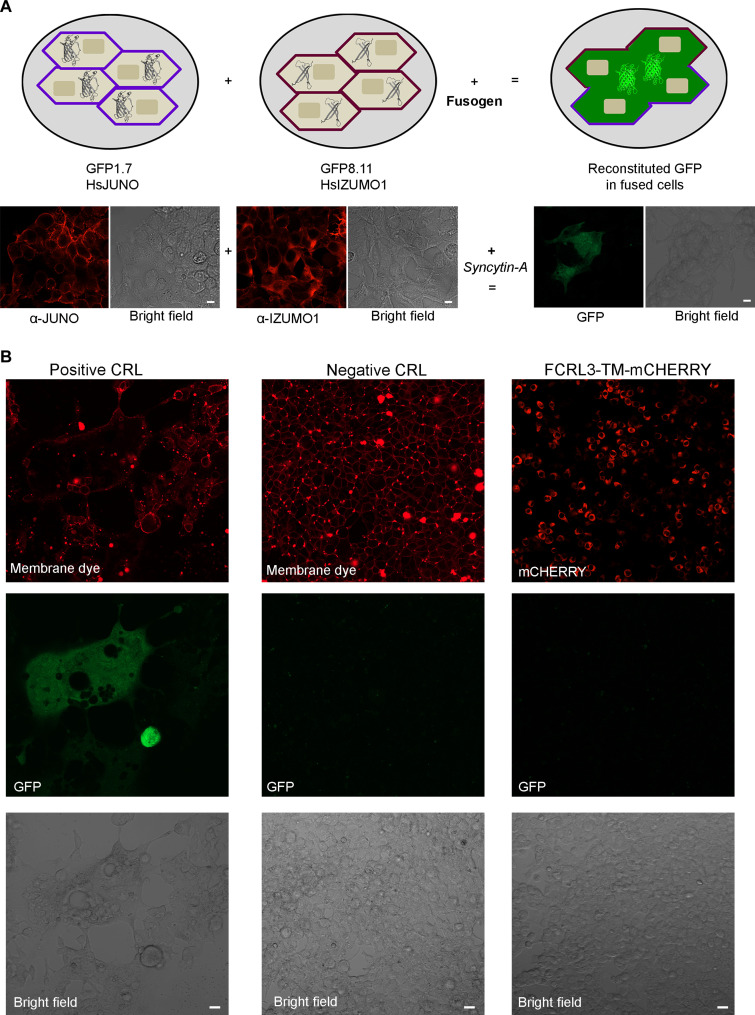
FCRL3 does not induce cell fusion between cells expressing either IZUMO1 or JUNO. (**A**) Two populations of cells, one expressing JUNO and the N-terminal GFP fragment 1–7, and the other expressing IZUMO1 and the C-terminal GFP fragment 8–11, were mixed and, 24 hours later, transfected with plasmids encoding the known fusogen Syncytin-A or FCRL3-TM-mCherry. Scale bars, 10 μm. (**B**) Cells expressing Syncytin-A formed large syncytia that were fluorescent, demonstrating GFP complementation as a result of cell fusion and cytoplasmic mixing (left). Mock transfection of cells did not induce membrane fusion, and no GFP fluorescence was detected (middle). Expression of the FCRL3-TM-mCherry protein was confirmed by red fluorescence, but no cellular fusion was observed (right). Where indicated, cell membranes were stained with a membrane dye to distinguish formation of syncytia from single cells. Scale bars, 20 μm.

Although we were unable to detect direct binding between FCRL3 and either IZUMO1 or JUNO, there is evidence that FCRL3 induces cell fusion between cells that express JUNO and sperm (*30*). To determine whether we could establish a role for FCRL3 in cell fusion, we used a cellular fusion assay based on a split GFP complementation assay using cells stably expressing either full-length IZUMO1 and the C-terminal (8–11) GFP fragment or JUNO and the N-terminal (1–7) fragment (*16*). The two cell populations were mixed together, transfected 24 hours later with the plasmid encoding for the putative fusogen, and cell fusion was observed by GFP fluorescence caused by protein complementation due to cytoplasmic mixing. Transfecting cells with the established fusogen *Syncytin-A* resulted in the formation of large syncytia that were fluorescent (Fig. 3), as has been previously reported (*16**,* *37*). By contrast, transfecting cells with a mammalian expression plasmid encoding a full-length FCRL3-TM-mCHERRY protein did not induce cell fusion, and no green fluorescence was observed (Fig. 3). Together, these data do not support a role for FCRL3 in sperm-egg recognition or fusion.

## DISCUSSION

Understanding the molecular mechanisms of fertilization is of particular interest in the field of reproduction because it will lead to the development of new diagnostics and treatments for infertile couples as well as improved approaches to contraception. The role of cell surface proteins and their interactions is especially important to understand because it provides a molecular explanation of how the haploid sperm and egg recognize each other and subsequently fuse their membranes to form a diploid zygote. A growing number of cell surface and secreted proteins have been identified by using targeted gene disruption in mice with the phenotype of sex-specific infertility despite morphologically normal sperm or eggs. Eight genes that result in male infertility (*IZUMO1, SPACA6, TMEM95, TMEM81, SOF1, FIMP*, *DCST1*, and *DCST2*) and an additional two that cause infertility in females (*JUNO* and *CD9*) have been identified. Of the proteins encoded by these genes, only IZUMO1 and JUNO have been shown to directly interact and are expressed by different gametes; the interaction appears to have an adhesive but no fusogenic role (*6*). While there is growing evidence that IZUMO1 functions in the context of a trimeric complex involving SPACA6 and TMEM81 (*22**,* *26*), how all these proteins could lead to membrane fusion remains mysterious. The recent discovery that FCRL3/MAIA interacted with both IZUMO1 and JUNO suggested the possibility that it could act as a bridging molecule with a potential role in membrane fusion during fertilization (*30*). Fusogenic roles have been reported for structurally similar members of the Ig superfamily such as FGFRL1 (*38*). It was not possible to genetically test the role of *FCRL3* in mice because mice lack a direct ortholog of this gene, although mice with a targeted deletion in a paralogous gene, *Fcrl5*, did show a reduction in fertility (*30*).

Here, we have attempted to further characterize the binding interactions of FCRL3 with IZUMO1 and JUNO, but were unable to find any evidence of direct interactions with either protein. We have used a protein interaction assay that was specifically developed to detect low-affinity extracellular protein interactions (AVEXIS). Although this assay is performed in vitro and uses recombinant proteins (Table 1), it is designed to detect direct interactions with equilibrium binding constants within the low micromolar range, as is typical of this class of interactions (*32*). We were also unable to detect interactions when FCRL3 was presented in the context of a cell membrane. Vondrakova *et al.* (*30*) observed some cell fusion events with human embryonic kidney (HEK) 293 cells transfected with FCRL3 and intact human sperm spermatozoa. This suggests that FCRL3 may have a role in gamete fusion even if it does not directly interact with IZUMO1. Last, we were also unable to detect any role of FCRL3 in an assay of cellular fusion, questioning the role that FCRL3 might play in fertilization.

Our data are consistent with several other observations. First, preincubating oocytes with an anti-JUNO monoclonal antibody blocks all IZUMO1 binding, suggesting that JUNO is the only IZUMO1 receptor at the oocyte surface (*6*). Second, two independent large-scale systematic protein interaction screens between proteins belonging to the human Ig superfamily did not report a direct interaction between IZUMO1 and FCRL3 (*39**,* *40*). Last, pairwise structural complex modeling of all the known extracellular proteins involved in fertilization including FCRL3 did not report any support for interactions involving FCRL3 (*26*). In summary, using methods that have been previously successful in identifying functionally relevant extracellular protein interactions, we were unable to obtain any evidence for direct interactions between FCRL3 and both IZUMO1 and JUNO, nor a role for FCRL3 in cell fusion.

## MATERIALS AND METHODS

### Cell culture and recombinant protein expression

Soluble recombinant proteins were produced by transient transfection of HEK293-6E mammalian cells grown in Gibco FreeStyle 293 expression medium (catalog no. 12338026, Thermo Fisher Scientific) as previously described (*41**,* *42*). Prey proteins were produced by transfecting 0.5 μg of DNA per million cells, and the conditioned medium was harvested 5 days after transfection. Prey protein expression levels were normalized using their β-lactamase activity to levels required for the AVEXIS assay, essentially as described (*41*). For the expression of biotinylated bait proteins, the bait plasmid was cotransfected with a plasmid encoding a secreted version of the biotin-ligase (BirA) (*43*) and dialyzed overnight in phosphate-buffered saline (PBS) to remove the unconjugated d-biotin in the medium. Baits were normalized by a standard ELISA using a monoclonal antibody (OX68) recognizing the Cd4d3+4 tag (*32*).

### Monoclonal antibody ELISA

Streptavidin-coated 96-well plates (436014, Thermo Fisher Scientific) were blocked in 2% bovine serum albumin (BSA; A9647, Sigma-Aldrich) in PBS–0.1% Tween 20 (PBS-T) for 1 hour at room temperature. A 1:2 dilution series of biotinylated FCRL3 or control protein (rat CD200R) was prepared in 2% BSA PBS-T, and 100 μl of the protein dilution was transferred to the blocked streptavidin-coated 96-well plate. To assess the presence of conformational epitopes, one-half of the protein sample was denatured by heating at 80°C for 10 min. After capturing the biotinylated proteins for 1 hour at room temperature, the plates were washed 3× with PBS-T. The monoclonal antibodies, anti-FCRL3 and anti-rat CD4, were added at the concentration of 1.4 μg/ml. The anti-rat Cd4 domain 3+4 monoclonal antibody (OX68) was raised against the tags of our recombinant proteins and used as a control. After 1 hour of incubation with the primary antibody and 3× PBS-T washes, a goat anti-mouse IgG (A9316, Sigma-Aldrich) conjugated to alkaline phosphatase was diluted 1:10,000 and incubated for 1 hour at room temperature. The plates were washed 3× with PBS-T, and 100 μl of phosphatase substrate *p*-nitrophenylphosphate (2 mg/ml) (P4744, Sigma-Aldrich) dissolved in a diethanolamine buffer (10% diethanolamine, 0.5 mM MgCl_2_, 10 mM NaN_3_, pH 9.8) was added to each well. Absorbance was measured on a Tecan Spark plate reader at 405 nm after 40 min.

### AVEXIS and SAVEXIS binding assays

The AVEXIS assay was performed as described elsewhere (*41*) with normalized baits and preys, as follows. Biotinylated bait proteins were immobilized in 96-well streptavidin-coated plates (734-1284, VWR) by incubating 100 μl for 1 hour at room temperature. Protein activities were measured with a mouse anti-rat Cd4 antibody (OX68) followed by incubation with an anti-mouse alkaline phosphatase secondary antibody and detected by measuring the hydrolysis products of the phosphatase substrate *p*-nitrophenylphosphate by absorbance at 405 nm. The bait protein activity used in the AVEXIS assay corresponded to the highest dilution for which saturation of the microtiter plate well was observed. To determine the concentration of preys to be used in each well, 60 μl of the chromogenic β-lactamase substrate nitrocefin (484400, Calbiochem) was added to 20 μl of prey proteins and the absorbance of the hydrolysis products was measured at 485 nm over 20 min. The concentration that resulted in a complete hydrolysis of the nitrocefin substrate within 4 to 10 min was selected. Bait proteins were captured on streptavidin-coated plates and washed three times in PBS-T. One hundred microliters of prey proteins was added and incubated for 1 hour at room temperature. Following three more washes in PBS-T and one wash in PBS, 60 μl of 242 μM nitrocefin was added, and the absorbance of the hydrolysis products was measured by light absorption at 485 nm with a Tecan Spark plate reader. Data were analyzed with Prism GraphPad software. The biotinylated protein consisting of the rat Cd4d3+4 tag alone was used as a negative control, while the binding of rat Cd200R to its ligand, rat Cd200, was used as a positive control.

The SAVEXIS assay was developed and performed as previously described (*35*). Soluble recombinant biotinylated ectodomains were secreted by transient transfection of the HEK293-6E cell line cultured in suspension. Proteins were purified from 25 ml of spent culture medium with Ni^2+^–nitrilotriacetic acid (Qiagen) resin by column method under native conditions according to the manufacturer’s instructions. Briefly, disposable columns containing resin were spun to remove storage solutions and washed with binding buffer (PBS, 40 mM imidazole, pH 7.4). Supernatants were loaded onto beads and washed three times with binding buffer before being eluted in 400 μl of elution buffer (PBS, 250 mM imidazole, pH 7.4). Streptavidin-coated 96-well plates (Thermo Fisher Scientific, 436014) were washed once in HEPES-buffered saline(HBS) with 0.1% Tween 20 (HBS-T), blocked in 2% BSA in HBS for 30 min at room temperature, and incubated with monomeric baits at a concentration of 13.4 nM for 16 hours at 4°C. After washing the plates three times with 150 μl of HBS-T supplemented with 0.8 μM desthiobiotin (Sigma-Aldrich, D1411), multimeric preys were added to the plates and incubated for 1 hour at room temperature. Multimeric preys were used at a final concentration of 4.4 nM and prepared by mixing the purified proteins with streptavidin–horseradish peroxidase (HRP) (Pierce, 21130) at 1.7 μg/ml. Immediately after three washes, 60 μl of tetramethylbenzidine chromogenic substrate (Millipore ES001) was added and incubated for 20 min. The absorbance was then measured on a Tecan Spark plate reader at 650 nm.

### Cell fusion assay

A complementation assay based on the spontaneous reassembly of two GFP fragments was performed as previously described (*16*). In brief, two stably transfected HEK-293T cell lines were generated, one expressing human JUNO and the GFP fragment 1–7, and the other one expressing human IZUMO1 and the GFP fragment 8–11. The cDNA encoding for human *IZUMO1* was excised from the open reading frame (ORF) clone RC206671 (OriGene Technologies Inc.) and ligated in the pIRESPuro3 vector (Clontech). The cDNA encoding for human *JUNO* was excised from a custom-made ORF clone (OriGene Technologies Inc.) (*33*) and ligated into the pIRESPuro3 vector (Clontech). Stable clones were obtained by selection with puromycin (10 μg/ml) and single cell sorting after transfection (*16*). The display of JUNO and IZUMO1 on the cell surface was confirmed by staining with IZUMO1 and JUNO soluble recombinant pentamers (*16*). To perform the cell fusion assay, an equal number of IZUMO1-spGFP_8–11_ and JUNO-spGFP_1–7_ cells were mixed, seeded in μ-Slide 8 Well High (80806, Thistle Scientific), and transfected 24 hours later with a mammalian expression plasmid encoding the putative fusogen. After 1 day, the presence of fused green cells was recorded using a Zeiss LSM980 laser scanning confocal microscope. Before imaging, some samples were stained with CellBrite Fix 640 (Biotium) following the manufacturer’s instructions.

### Cell-based binding assay and immunostaining

HEK293-6E cells growing in suspension were transfected with plasmids encoding the entire ectodomains of human FCRL3, human JUNO, or human IZUMO1 tagged with the sequences encoding for the rat CD200R transmembrane domain followed by enhanced GFP (EGFP). Cells were transiently transfected with PEI (polyethylenimine “Max”) using a 3:1 ratio with DNA and maintained in culture for 24 hours. One day after transfection, equal numbers of cells were incubated for 1 hour at 37°C with proteins clustered around a streptavidin-phycoerythrin conjugate (405204, BioLegend) as previously described (*44*), washed with Dulbecco’s PBS (DPBS), fixed with 4% formaldehyde, and mounted on a microscope glass slide. For the detection of FCRL3, cells were incubated with anti-FCRL3 monoclonal mouse antibody (MAB3126, R&D Systems), diluted 1:100 in PBS–2% BSA, washed briefly in PBS, fixed with a 4% formaldehyde solution (obtained from 16% formaldehyde, methanol-free, 28906, Thermo Fisher Scientific) for 20 min, and then incubated for 1 hour with an anti-mouse Alexa Fluor 568–conjugated secondary antibody (Molecular Probes). Last, nuclei were stained with Hoechst 33342 (SKU: 639, Immunochemistry Technologies LLC), and images were acquired by confocal microscopy with a Zeiss LSM980 microscope. IZUMO1-spGFP8-11 and JUNO-spGFP1-7 cells, seeded in a multiwell microslide with glass bottom (IB-81817, Thistle Scientific Ltd.), were stained with Nanobodies (a gift from J. Lee) raised against the entire ectodomains of human IZUMO1 and human JUNO, respectively. The cells were fixed with 4% formaldehyde in DPBS and incubated with anti-JUNO or anti-IZUMO nanobody (5 μg/ml) for 1 hour at room temperature, washed with DPBS, and incubated with anti-alpaca IgG (1 μg/ml) (VHH domain) (Jackson ImmunoResearch) and finally with an Alexa Fluor 594–conjugated anti-goat secondary antibody (Molecular Probes) diluted 1:500 in DPBS. All images were acquired with a confocal Zeiss LSM980 microscope.
